# Human monocyte-to-macrophage differentiation involves highly localized gain and loss of DNA methylation at transcription factor binding sites

**DOI:** 10.1186/s13072-019-0279-4

**Published:** 2019-06-06

**Authors:** Koen F. Dekkers, Annette E. Neele, J. Wouter Jukema, Bastiaan T. Heijmans, Menno P. J. de Winther

**Affiliations:** 1 Molecular Epidemiology, Department of Biomedical Data Sciences, Leiden University Medical Center, Leiden, The Netherlands; 2 Department of Medical Biochemistry, Amsterdam Cardiovascular Sciences, Meibergdreef 9, Amsterdam UMC, University of Amsterdam, Amsterdam, The Netherlands; 3 Department of Cardiology, Leiden University Medical Center, Leiden, The Netherlands; 4 Institute for Cardiovascular Prevention (IPEK), Munich, Germany

**Keywords:** DNA methylation, Differentiation, Epigenomics, Macrophage, Monocyte, Transcription factor

## Abstract

**Background:**

Macrophages and their precursors monocytes play a key role in inflammation and chronic inflammatory disorders. Monocyte-to-macrophage differentiation and activation programs are accompanied by significant epigenetic remodeling where DNA methylation associates with cell identity. Here we show that DNA methylation changes characteristic for monocyte-to-macrophage differentiation occur at transcription factor binding sites, and, in contrast to what was previously described, are generally highly localized and encompass both losses and gains of DNA methylation.

**Results:**

We compared genome-wide DNA methylation across 440,292 CpG sites between human monocytes, naïve macrophages and macrophages further activated toward a pro-inflammatory state (using LPS/IFNγ), an anti-inflammatory state (IL-4) or foam cells (oxLDL and acLDL). Moreover, we integrated these data with public whole-genome sequencing data on monocytes and macrophages to demarcate differentially methylated regions. Our analysis showed that differential DNA methylation was most pronounced during monocyte-to-macrophage differentiation, was typically restricted to single CpGs or very short regions, and co-localized with lineage-specific enhancers irrespective of whether it concerns gain or loss of methylation. Furthermore, differentially methylated CpGs were located at sites characterized by increased binding of transcription factors known to be involved in monocyte-to-macrophage differentiation including C/EBP and ETS for gain and AP-1 for loss of methylation.

**Conclusion:**

Our study highlights the involvement of subtle, yet highly localized remodeling of DNA methylation at regulatory regions in cell differentiation.

**Electronic supplementary material:**

The online version of this article (10.1186/s13072-019-0279-4) contains supplementary material, which is available to authorized users.

## Background

Inflammation is characterized by the recruitment of monocytes upon migration from the blood differentiated into macrophages [1]. These cells participate in many aspects of inflammation such as host defense, tissue remodeling and wound healing [2]. Due to their broad range of functional capacities, macrophages are important regulators of disease outcome. Local environmental triggers can induce different activation states of macrophages. In vitro, cells treated with lipopolysaccharide (LPS) plus interferon gamma (IFNγ) or interleukin-4 (IL-4) are at the two extreme ends of the macrophage activation spectrum, with the first having pro-inflammatory characteristics and the latter being considered anti-inflammatory [3]. Foam cells (i.e., macrophage that have engulfed modified lipids in the arterial wall) play an important role in the development of atherosclerosis, the primary cause of cardiovascular disease, which can result in a myocardial infarction or stroke [4]. Understanding the molecular mechanisms controlling macrophage differentiation and activation will aid in understanding their functioning in health and disease.

Differentiation and activation processes of macrophages are accompanied by pronounced epigenetic remodeling to accommodate the changes in their transcriptional repertoire [5]. DNA methylation is an essential component of the epigenome and defines cell identity. It occurs predominantly at CpG sites and previous studies identified loss of methylation as being dominant during monocyte-to-macrophage differentiation [6, 7]. While loss of methylation at promoter regions is traditionally thought to increase gene expression, recent data challenge this view indicating that both gain and loss of methylation can be associated with increased transcriptional activity in particular at non-promoter regions [8, 9]. What is known is that regions of dynamic DNA methylation between different cell types co-localize with enhancers and transcription factor binding sites [10], in line with a role of transcription factors in the methylation process. Recent studies showed that changes in DNA methylation can be both a cause [9] and consequence [11] of transcription factor binding and may also be involved in stabilizing regulatory states [12]. So while the exact molecular role of DNA methylation appears to be context-specific, mapping dynamic DNA methylation may be instrumental in identifying the regulatory regions in the genome that control macrophage differentiation and activation.

Here, we investigated both genome-wide and whole-genome DNA methylation changes in monocyte-to-macrophage differentiation and subsequent activation (LPS/IFNγ, IL-4, modified lipids). We found that not only loss but also gain of DNA methylation is common during macrophage differentiation. Of interest, such DNA methylation changes are highly localized, often affecting single CpGs only, and primarily located in enhancer regions defined by specific transcription factor binding sites. Interestingly, we found that gain of methylation, a previously ignored phenomenon in monocyte–macrophage differentiation, was associated with increased binding of lineage determining TFs.

## Results

### Marked DNA methylation changes occur during monocyte-to-macrophage differentiation but not upon subsequent macrophage activation

**Fig. 1:**
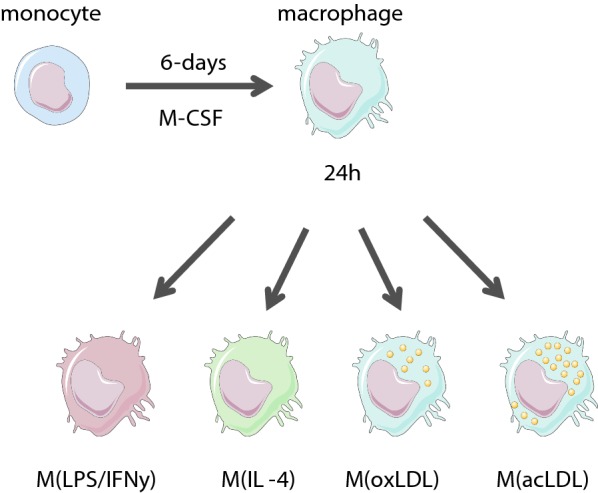
Study design. Monocytes were isolated from four healthy donors and differentiated to macrophages in the presence of MCSF. Macrophages were subsequently activated to obtain different macrophage subsets

Monocytes were isolated from four healthy donors and differentiated to macrophages in vitro. They were subsequently activated with LPS/IFNγ, IL-4, oxidized low-density lipoprotein (oxLDL) or acetylated LDL (acLDL) to obtain different activation states. An overview of the study design and a summary of the primary characterization of the cells are shown in Fig. 1 and Additional file 1: Figure S1.

We evaluated DNA methylation during differentiation and further activation at 440,292 CpGs. Samples clustered primarily on sex (principal component 1, 37% variance explained), donor (principal component 2 and 3, 17% and 16% variance explained, respectively), in line with the genetic component of DNA methylation [8, 13] and also on monocyte versus macrophage differentiation (principal component 4, 6% variance explained) (Additional file 1: Figure S2). A paired analysis testing for differences within donors identified 5870 differentially methylated CpGs (DMCs) (*P*_FDR_ < 0.05, mean squares > 0.025) between the six cell types (Table 1 and Additional file 2: Table S2). The large majority of DMCs could be attributed to monocyte-to-macrophage differentiation (98%, 5780 DMCs, Table 1). In contrast to previous data (6), DMCs included both gain of DNA methylation (gain-DMCs, *n* = 4283) and loss of DNA methylation (loss-DMCs, *n* = 1497), with the first being most common. DMCs had a mean methylation level that was uniformly distributed from ~ 0 to ~ 100%, and likewise the difference between monocytes and macrophages was similar across methylation levels (mean difference in methylation: 13%, Additional file 1: Figure S3). However, some DMCs switched from hypomethylated to hypermethylated and vice versa during differentiation (Additional file 1: Figure S3).

**Table 1:** Summary of the primary results

Differentiation	DMCs	Gain	Loss
	5780	4283	1497

Activation	DMCs	Gain	Loss
M (LPS/IFNγ)	69	4	65
M (IL-4)	6	3	3
M (oxLDL)	4	0	4
M (acLDL)	3	0	3

Macrophage-specific	DMCs
	8

DNA methylation in all subsets was measured using the Illumina 450k array, and an epigenome-wide analysis was performed using a linear mixed model with donor as random effect

Shown are the amount of differentially methylated CpGs (DMCs) during differentiation and activation based on partial t-statistic and split by gain and loss of methylation (*P*_FDR_ < 0.05, mean squares > 0.0025)

Subsequent activation of macrophages with IL-4 or lipids resulted in fewer than < 10 CpGs, while 69 DMCs (4 gain, 65 loss) were found in response to activation with LPS/IFNγ (Table 1 and Additional file 2: Table S2). In addition, we identified a small number of DMCs where multiple macrophage types contributed to the difference in methylation: cg04739200 [macrophage and M (IL-4)], cg27000690 [macrophage and M (IL-4)], cg06850284 [macrophage and M (acLDL)], cg26933866 [macrophage and M (LPS/IFNγ)] and cg23248885 [M (oxLDL)] and M [(acLDL)] (Additional file 1: Figure S4).

To validate our results, we re-analyzed a public 450k data set of monocytes, macrophages and LPS-activated macrophages [7]. Despite differences in culture (G-MCSF vs. MCSF) and activation conditions (LPS vs. LPS + IFNγ), we observed a high correlation of effect sizes for the 5870 DMCs between the two data sets (*R*_Monocyte_ = 0.77, *R*_Macrophage_ = 0.63 and *R*_M(LPS/IFNγ_) = 0.72, Additional file 1: Figure S5).

### Genes linked to DMCs are enriched for processes involved in monocyte-to-macrophage differentiation and macrophage activation for both gain and loss of methylation

**Fig. 2:**
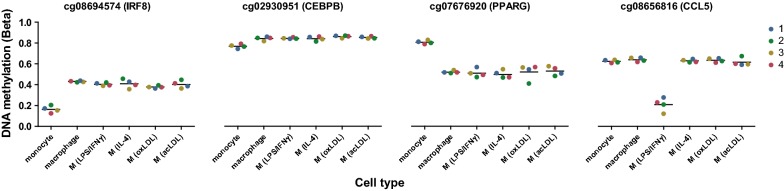
DMCs mapped to their nearest gene include hallmark examples of macrophage-related genes. DNA methylation beta values for DMCs near genes involved in monocyte-to-macrophage differentiation (*IRF8*, *CEPB*, *PPARG*) and for LPS/IFNγ macrophage activation (*CCL5*) for the four donors

To identify potential pathways affected by DNA methylation changes, we subsequently mapped the DMCs to their nearest genes (Additional file 2: Table S2). We observed that these genes included hallmark examples of genes involved in monocyte-to-macrophage differentiation for both gain-DMCs (e.g., *IRF8*, *CEBPB*) and loss-DMCs (e.g., *PPARG*) [14, 15] (Fig. 2). Additionally, we also found genes for LPS/IFNγ macrophage-specific activation (e.g., *CCL5*).

**Fig. 3:**
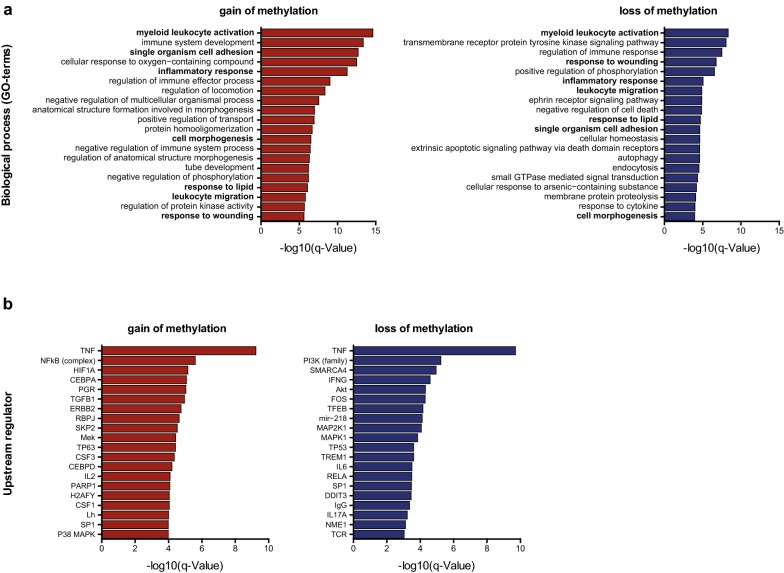
Genes linked to DMCs are enriched for processes involved in monocyte-to-macrophage differentiation for both gain and loss of methylation. Enrichment analysis for differential DMCs mapped to their nearest gene for gain and loss of DNA methylation. **a** Pathway analysis for GO terms biological processes. **b** Upstream regulator analysis by Ingenuity Pathway Analysis (IPA). Shown is the Top 20

Pathway analysis showed strong enrichment for processes involved in monocyte-to-macrophage differentiation both for genes linked to gain-DMCs (2152 unique genes) and for genes linked to loss-DMCs (771 unique genes) (Fig. 3a). Of interest, the top 20 enriched pathways for gain-DMC-associated genes had seven processes in common with the top 20 enriched pathways for loss-DMC-associated genes including ‘myeloid leukocyte activation,’ ‘single organism cell adhesion’ and ‘inflammatory response’ (Fig. 3a). Using public RNA-seq data, we found that gain of methylation was associated with a reduced transcription of the nearest genes (*P* value = 0.04) while loss of methylation was associated with increased transcription (*P* value = 2.6 × 10^−6^), a trend that was also observed for the genes in the seven overlapping pathways (Additional file 1: Figure S6).

Further analysis showed that gain and loss of methylation also shared their upstream regulator, tumor necrosis factor (TNF), a key inflammatory cytokine and known regulator of monocytes and macrophages (Fig. 3b). Together, these data imply that both gain and loss of DNA methylation are functionally important in monocyte-to-macrophage differentiation and suggest an involvement of TNF responses in methylation changes during differentiation.

For completeness, we performed a pathway analysis for the nearest genes of 65 loss-DMCs during LPS/IFNγ macrophage-specific activation, which revealed enrichment for metal ion homeostasis, positive regulation of GTPase and cellular response to interferon gamma (Additional file 1: Figure S7).

### Differential methylation during monocyte-to-macrophage differentiation preferentially occurs at enhancers

**Fig. 4:**
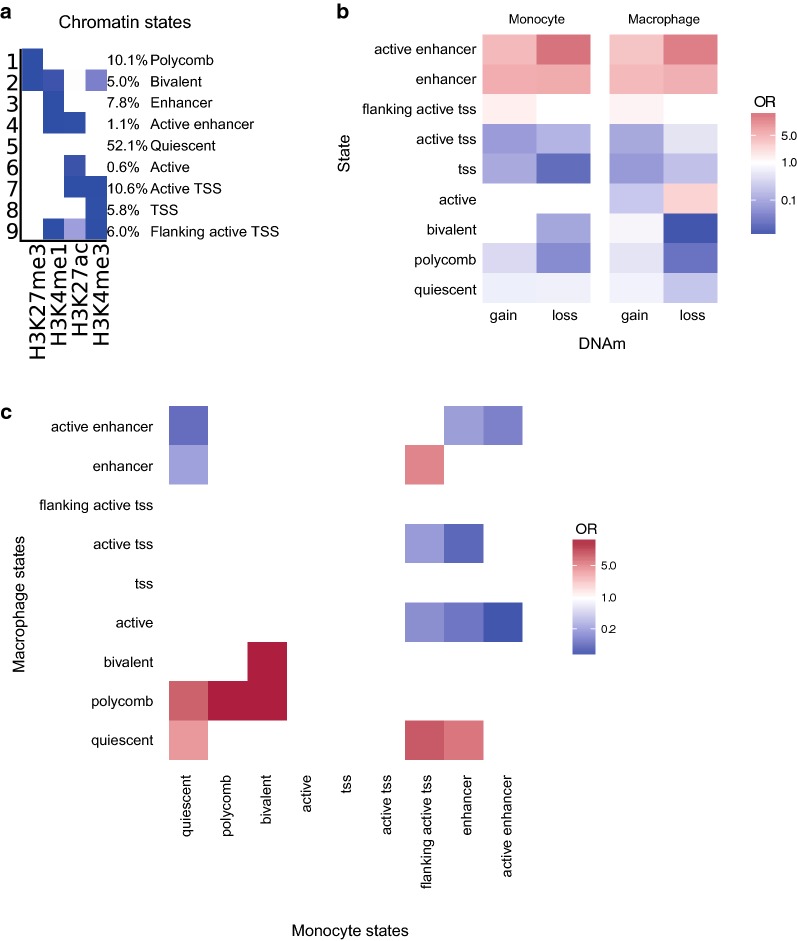
Chromatin states at DMCs are enriched for enhancers, and loss of methylation is enriched for regions that become more active during differentiation. **a** Emission parameters of the nine chromatin states learned using ChromHMM. **b** Enrichment analysis of DMCs based on chromatin states for monocytes (left) and macrophages (right) for gain and loss of methylation compared to genome-wide chromatin states. **c** Enrichment of gain over loss for each of the transitions from monocyte (*x*-axis) to macrophage (y-axis). **b**, **c** are plotted in log2 scale

To characterize the regulatory landscape at the DMCs, we defined nine chromatin states in monocytes and macrophages seperately using a hidden Markov model based on H3K4me1, H3K4me3, H3K27ac and H3K27me3 histone marks available from public BLUEPRINT data (Fig. 4a, Additional file 3: Table S3). DMCs associated with monocyte-to-macrophage differentiation were enriched for enhancers (H3K4me1) and active enhancers (H3K4me1 + H3K27ac) in both monocytes and macrophages (*P*_FDR_ < 0.05) (Fig. 4b). Strikingly, this was not only the case for loss-DMCs (OR > 5.6), but also for gain-DMCs (OR > 3.8). Transcription start sites (TSS, H3K4me3) and repressed states (quiescent (none), polycomb (H3K27me3) and bivalent states (H3K27me3 + H3K4me1 + H3K4me3) were underrepresented at DMCs in both cell types (OR < 1). The enrichment of DMCs at enhancers as inferred from chromatin states was confirmed by analyzing enhancers identified through promoter capture Hi-C [16] in monocytes (OR = 1.5, *P* value < 2.2 × 10^−16^) and macrophages (OR = 1.4, *P* value < 2.2 × 10^−16^).

A direct comparison of loss- versus gain-DMCs revealed that, as expected, gain-DMCs were relatively enriched in regions that remained repressed during monocyte-to-macrophage differentiation (i.e., quiescent, polycomb or bivalent; OR > 3.11 & *P*_FDR_ < 0.05) or that acquired a repressed state in macrophages while being an enhancer or flanking active TSS in monocytes (OR = 5.9 and 11.5, Fig. 4c). Conversely, loss-DMCs were generally enriched for regions that either lose a repressive or acquire a more active chromatin state during monocyte-to-macrophage differentiation (e.g., quiescent regions becoming enhancers (OR = 3.4) and activation of enhancers (OR = 4.9).

### DNA methylation changes are located at single CpGs or small regions that are enriched for changes DNAseI hypersensitive sites and specific transcription factor binding sites

**Fig. 5:**
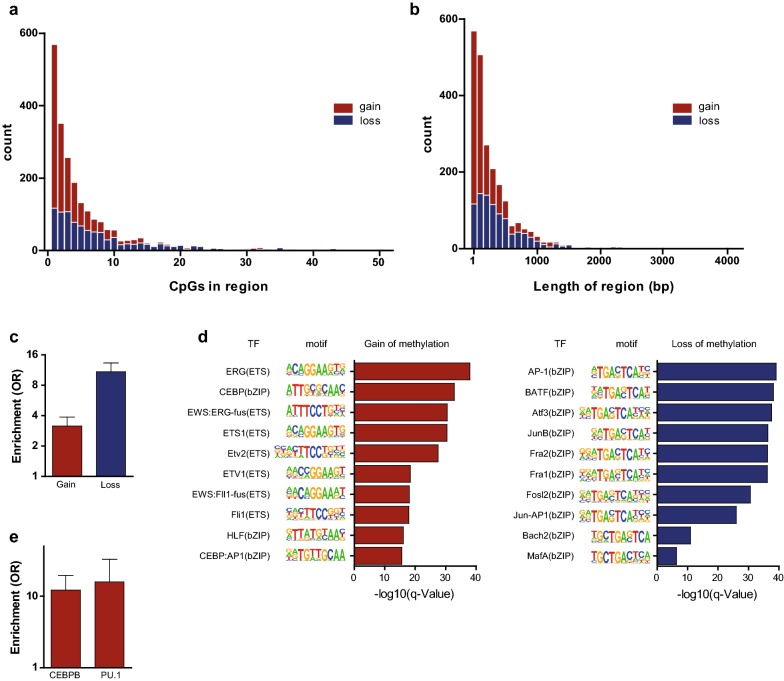
DNA methylation changes are located in small regions which are enriched for changes in DNAseI hypersensitive sites and TF-binding sites. **a** Histogram depicting the number of DMRs harboring a specific amount of CpGs. **b** Histogram depicting the number of DMRs having similar length of region in base pairs. **c** Enrichment analysis for DNAseI hypersensitive sites for gain (red) and loss (blue) of DNA methylation compared to genome-wide DNAseI hypersensitive sites. **d** Motif analysis with HOMER for transcription factor binding sites on differential DMCs for gain (left) and loss (right) of DNA methylation. **e** Enrichment analysis of transcription factor binding of C/EBP and PU.1 at their motifs for gain-DMCs during monocyte-to-macrophage differentiation

To assess whether or not DMCs identified using the sparse 450k array represented differentially methylated regions (DMRs), we overlaid DMCs with public whole-genome bisulfite sequencing data of monocytes and macrophages [17]. Of the 5870 DMCs, 4600 CpGs were sufficiently covered in the WGBS data. For the 4600 CpGs, the difference in methylation between monocytes and macrophages was generally similar in the two data types (*R* = 0.77, Additional file 1: Figure S8). 2213 of the CpGs also met the threshold of a ≥ 5% methylation difference in the same direction in WGBS, a 10.7-fold enrichment as compared with non-DMCs (*P* value < 2.2 × 10^−16^). Surprisingly, for 26% of these CpGs, the methylation difference did not extend to neighboring CpGs and the differential methylation remained confined to a single CpG (Fig. 5a). The median number of differentially methylated CpGs was 3, and consequently, the length of DMRs was generally short (median 112 bp, Fig. 5b). We hypothesized that such highly localized differences were associated with regulatory regions. Indeed, we found that the DMCs were enriched for changes in DNAseI hypersensitive sites during monocyte-to-macrophage differentiation, a localized mark (~ 150 bp) of open chromatin often colocalizing with transcription factor (TF) binding [18]. This was true for both CpGs that gained (OR = 2.3, *P* value < 2.2 × 10^−16^) and lost (OR = 8.4, *P* value < 2.2 × 10^−16^) methylation during differentiation (Fig. 5c). Subsequent motif analysis of the DMCs involved revealed that gain-DMCs were enriched for binding sites of the TFs ETS and C/EBP, while loss-DMCs were enriched for bZIP motifs and contained mainly AP-1 TFs (AP-1, ATF3, JUNB) (Fig. 5d), all TFs that are known to play a role in monocyte-to-macrophage differentiation. Strikingly, an analysis of ChIP-seq data revealed that gain of methylation at motifs of C/EBP and the ETS transcription factor PU.1 was associated with increased binding of these TFs (C/EBP: OR = 12.2, *P* value = 1.0 × 10^−7^, mean methylation difference = 28.1%; PU.1: OR = 15.9, *P* value = 1.4 × 10^−15^, mean methylation difference = 27.7%, Fig. 5e).

### Subjective cutoffs can obscure functionally relevant localized and gains of methylation

**Fig. 6:**
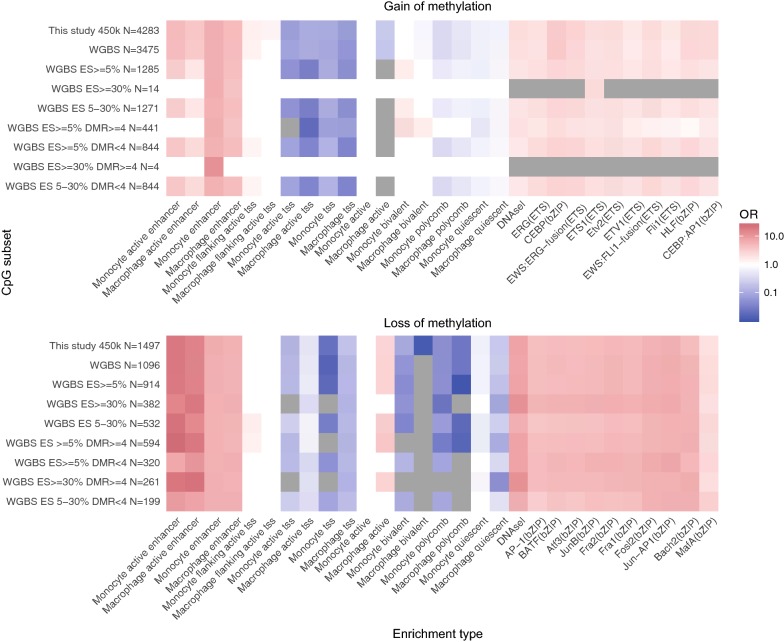
Arbitrary, stringent thresholds can lead to an incomplete and biased view of DNA methylation remodeling during monocyte–macrophage differentiation. Enrichments for chromatin segments, DNAseI hypersensitive sites and top 10 binding sites of macrophage TFs were calculated for gain-DMCs and for loss-DMCs for all DMCs identified in this study with different thresholds. For effect size cutoffs and DMRs, we used DMCs sufficiently measured in WGBS data. For the DMCs with consistent direction, we used effect size cutoffs of > 5%, between 5 and 30% or > 30%. Enrichment analysis for different effect size cutoffs was plotted separately or in combination with DMR thresholds: ≤ 4 or > 4

Previous studies primarily reported on loss of DNA methylation during monocyte-to-macrophage differentiation (6, 7), whereas we also identified substantial gain of methylation. We hypothesized that this was due to differences in analyses, in particular the choice for more stringent effect size cutoffs (10 or 30% difference in methylation versus 5% in our analysis) and the choice to focus on identifying DMRs covering at least 4 CpGs instead of individual DMCs. We therefore split our DMCs in subsets based on these two thresholds (30% difference in methylation and ≥ 4 CpGs). As expected, a substantial proportion of DMCs was lost after applying these stringent thresholds: 82.0% of DMCs when restricting DMCs to those with a > 30% difference, 52.9% when restricting to DMRs of at least 4 CpGs, and 87.9% of DMCs were lost when both thresholds were applied simultaneously (Fig. 6). Inspection of gain- and loss-DMCs separately, however, indicated that the thresholds resulted in the exclusion of nearly all gain-CpGs (99.7%).

We next performed the functional enrichment analyses we applied previously (i.e. chromatin segmentation, DNAseI hypersensitive sites and TF motifs) for DMCs surviving the different thresholds. Strikingly, enrichments were hardly affected provided that sufficient DMCs remained available for analysis (Fig. 6). This observation indicates that while gain of methylation co-localizes with the same functionally relevant genomic regions as loss of methylation, its occurrence was previously missed due to the application of stringent yet arbitrary thresholds. An example is the gain-DMC cg01059398 which is located in exon 2 of *TNFSF10* (Additional file 1: Figure S9). The DMC did not extend to neighboring CpGs in WGBS data, but mapped to an enhancer with a PU.1 binding site in both monocytes and macrophages, that becomes a DNAseI hypersensitive site and acquires CEBP binding during macrophage differentiation.

## Discussion

We here report on dynamic DNA methylation during monocyte-to-macrophage differentiation and subsequent activation. We show that DNA methylation occurs primarily during monocyte-to-macrophage differentiation and substantially less during macrophage activation. Dynamic methylation is not restricted to loss of methylation as previously reported but commonly involves gain of methylation. Surprisingly, genomic annotations of regions acquiring gain and loss of methylation were similar and both were enriched for enhancers. Our data further reveal that remodeling of the epigenome during differentiation predominantly involves very local tweaking of the methylation status and not changes across larger regions. We found that these local changes are associated with binding sites of transcription factors responsible for macrophage identity including ETS and C/EBP for gain of methylation, and bZIP motifs and AP-1 factors for loss of methylation. Our data imply that localized gains and losses of methylation are associated with specific changes in epigenomic regulation that play an important role in the differentiation process.

While previous studies primarily observed loss of methylation during monocyte-to-macrophage differentiation [6, 7], we predominantly observe gain of DNA methylation. We found that both gain and loss of methylation are located near genes involved in monocyte-to-macrophage differentiation and macrophage activation indicating that both gain (associated with reduced transcription of nearest genes) and loss (associated with increased transcription of nearest genes) of methylation are biologically relevant. In line with the latter, we found that these genes were enriched for pathways involved in cell morphogenesis, leukocyte migration, inflammatory response, myeloid leukocyte activation, response to lipid, response to wounding and single organism cell adhesion for both gain and loss of methylation. TNF, a pro-inflammatory cytokine secreted in response to activation of monocytes and macrophages and an important regulator of macrophage function [3], was predicted as a primary upstream regulator for both gain and loss in DNA methylation. The fact that TNF is associated with gain and loss of methylation suggests that there is a rewiring of the TNF response upon monocyte-to-macrophage differentiation through alterations in epigenomic regulation.

While loss of methylation has previously been observed particularly at enhancer regions, we report that this is also true for gain of methylation. Nevertheless, gain of methylation mostly occurred at regions that become more repressed during monocyte-to-macrophage differentiation. Conversely, loss of methylation occurs at regions that become more active, consistent with previous studies where similar effects were observed in B-cell differentiation [19] and in fetal development [20].

A key question in the field is whether DNA methylation changes across regions (encompassing multiple CpGs) are biologically more relevant than changes at individual CpGs [21]. We observed that methylation changes occur mainly at single CpG sites or small regions. These sites are enriched for changes in DNAseI hypersensitive sites. As a consequence, studies that set out to find differentially methylated regions (DMRs), for example the study that reported differential methylation between monocytes and macrophages at DMRs (> 4 CpGs) [6], are expected to miss a substantial fraction of the DNA methylation changes, in particular at enhancers which are generally CpG poor [22]. Similarly, the choice of effect size cutoff plays a crucial role in DNA methylation studies. We found that the observation in previous studies that loss of methylation dominates monocyte-to-macrophage differentiation may be an artifact of an arbitrary, stringent effect size threshold. Our analysis showed that DMCs with smaller effect sizes (5-30% difference in methylation) and/or occurring as single DMCs or part of a small DMC (< 4 CpGs) were likewise enriched for enhancers, DNAseI hypersensitive sites and similar TF-binding sites. Hence, gain and loss of methylation may be equally relevant for understanding differentiation. The use of seemingly conservative cutoffs that lack a compelling biological foundation will lead to a biased and incomplete view of epigenomic remodeling during cell differentiation. Complex functional studies would be required to derive biologically relevant thresholds. This finding may be equally relevant for the analysis and interpretation of epigenome-wide association studies of environmental exposures and disease traits.

We report that DMCs for both gain and loss of methylation are enriched at binding sites for TFs known to control macrophage function. Gain of methylation is enriched for binding sites of C/EBP and ETS (e.g., PU.1) TFs, while loss of methylation is enriched for binding sites for TFs with bZIP motifs, like AP-1 factors (AP-1, ATF3, JUNB). Some of the DMCs were outside the actual TF-binding motif. Although previous studies showed that both DNA methylation [8] and genetic variation [23] near but not in the motif can affect TF binding, our study cannot establish whether DMCs influence TF binding or are a passive marker of this phenomenon. Macrophage-specific enhancers are in general enriched for motifs that bind lineage determining transcription factors (LDTFs) determining cell identity [24]. In mouse macrophages, enhancers are enriched for motifs that bind PU.1 (ETS factor) and CEBP, which are required for the differentiation and function of macrophages [25, 26]. Motif analysis of the enhancers which overlapped with open chromatin identified the LDTFs PU.1 and C/EBPβ and C/EBPα as central regulators of myeloid enhancers [27]. Also human macrophage enhancers are enriched for C/EBP and PU.1 binding motifs [28]. Interestingly, we identified these two LDTFs to be associated with gain of methylation. In contrast, we found AP-1-like TFs to be strongly associated with loss of methylation. AP-1 like factors are TFs involved in differentiation but also in regulating macrophage activation and production of inflammatory factors such as cytokines and chemokines [14, 29].

Whether the DNA methylation changes we observe drive monocyte-to-macrophage differentiation or are a consequence of changes in (epigenomic) regulation or stabilize regulatory states [12] is an open question. The DNA methylation changes we observe may, for example, be the downstream effects of histone modifications or transcription factor binding [8, 30]. Moreover, feedback mechanisms may be at play in which occupancy of a transcription factor binding site inhibits local DNA methylation and vice versa methylation of that binding site inhibits transcription factor binding [31]. Systematic in vivo experiments need to be performed for each of these scenarios to unequivocally elucidate the biological situation.

## Conclusion

Epigenetic remodeling during monocyte-to-macrophage differentiation involves highly localized gain and loss of DNA methylation changes at binding sites of transcription factors.

## Materials and methods

### Monocyte isolation and macrophage culture

Peripheral blood mononuclear cells were isolated from four healthy donors [three males; mean age 31.5 (SD 4.7)] from buffycoats (Sanquin blood supply, Amsterdam, the Netherlands) through density centrifugation using Lymphoprep™ (Axis-Shield, Dundee, Scotland). Monocytes were purified using human CD14 magnetic beads and MACS^®^ cell separation columns (Miltenyi Biotec, Bergisch Gladbach, Germany). Monocytes were plated in 6-well tissue culture plates at a density of 1 × 10^6^ cells/mL for 45 min allowing monocyte adherence in Iscove’s Modified Dulbecco’s Medium (IMDM, Sigma-Aldrich, Zwijndrecht, The Netherlands) supplemented with 2 mM l-glutamine, penicillin (100 U/mL), streptomycin (100 µg/mL) and 1% fetal calf serum (FCS; All Gibco, Waltham, MA). Hereafter, monocytes were used for experiments or differentiated to macrophages by replacing the medium with IMDM plus 10% FCS and 50 ng/mL MCSF (Miltenyi Biotec, Bergisch Gladbach, Germany) for 6 days. On day 3, half the medium was removed and substituted by fresh IMDM with 10% FCS and 50 ng/mL MCSF. On day 6, all media were removed and replaced by IMDM with 10% FCS without MCSF and cells were activated by various stimuli for 24 h to gain different macrophage activation states: LPS/IFNγ (10 ng/mL, Sigma-Aldrich, Zwijndrecht, The Netherlands; 50 ng/mL R&D Systems, Minneapolis, MN), IL-4 (50 ng/mL, PreProTech, Rocky Hill, NJ), oxLDL (50 µg/mL Sanbio B.V., Uden, The Netherlands) and acLDL (50 µg/mL Sanbio B.V., Uden, The Netherlands) (Fig. 1).

### Flow cytometry

As a control for monocyte/macrophage purity and activation, we performed flow cytometry on all subsets. 0.2 × 10^6^ cells were blocked and stained with the following antibodies for purity and differentiation: CD14, CD16, HLA-DR, CCR5, CD68; LPS/IFNγ activation: CCR7 and CD64 or IL-4 activation: CD200R and CD206 (Additional file 1: Table S1). CD68 was stained intracellular after fixation and permeabilization following manufactures instruction (eBioscience, San Diego, CA). Fluorescence was measured with BD Canto II and analyzed with FlowJo software version 7.6.5. (FlowJo, LLC, Ashland, OR). Monocyte purity was based on CD14 + or CD16 + gating, and the expression of surface markers is presented as median fluorescence intensity (MFI).

### Oil red O staining

To visualize lipid uptake, cells were plated on coverslips and lipids were stained with Oil Red O staining (0.3% in 60% isopropanol, Sigma). Pictures were made with a Leica DM3000 microscope.

### DNA methylation

Genomic DNA was purified using the QIAamp DNA Blood Mini Kit (Qiagen, Hilden, Germany), bisulfite converted (500 ng) with the Zymo EZ DNA methylation kit (Zymo Research, Irvine, CA, USA) and hybridized (4 μl) on the Illumina 450K array using the manufacturer’s protocol (Illumina, San Diego, CA, USA). Data were generated by the Human Genotyping facility (HugeF) of ErasmusMC, The Netherlands.

### Quality control and normalization

For all samples, Illumina 450k array data passed quality control using MethylAid [32]. Sample mix-ups between donors were excluded on the basis of inferred genotypes using OmicsPrint [33]. Probes with detection *P* value > 0.01, bead number < 3 or zero intensity in at least one sample were removed (46718 probes were removed, resulting in a final data set of 440292 CpGs). Data were normalized using minfi’s [34] functional normalization [35] (five principal components). A workflow for the quality control and normalization pipeline is available at https://molepi.github.io/DNAmArray_workflow/index.html. Data to be submitted to EGA (EGAS00001003668).

### Statistical analyses

All statistical analyses were performed using R 3.4.1 [36]. The paired epigenome-wide analysis was performed on methylation beta values using a linear mixed model with donor as random effect for each CpG using the *lmer* and *aov* functions in *lme4* [37] with *P*-values calculated using Satterthwaite’s approximation [38]. Differentially methylated CpGs (DMCs) were obtained after adjusting for multiple testing using the Benjamini–Hochberg method and deciding on a mean-square cutoff of 0.0025; a threshold that would imply a 5% difference in methylation if only two conditions were considered. Principal components were obtained using the *prcomp* function in *stats* to visualize the characteristics of the DNA methylation data.

Nearest genes were found based on distance to the nearest transcription start or end site. Gene ontology enrichment was performed using Metascape [39] (only GO Biological Processes), and upstream regulators were found using Ingenuity Pathway Analysis (IPA) [40] (standard settings). Blueprint RNA-seq data [17] were downloaded for monocytes (donors: C000S5, C0010K, C0011I, C001UY and C004S) and macrophages (donors: C005VG, S001S7, S0022I and S00390) and transcription values (logTPM) were averaged for each cell type. Distributions of transcription levels were compared using a Wilcoxon signed-rank test.

Blueprint ChIP-seq peak files were downloaded for histon marks H3K4me1, H3K4me3, H3K27ac and H3K27me3 for 5 donors (C005VG, S001S7, S0022I, S00390 and S01F8K) with both monocyte and macrophage data [17]. Peak files were converted to a binary format (0 = no peak, 1 = peak), and ChromHMM [41] was used on these converted data to learn nine chromatin states (standard settings), which were labeled according to Roadmap reference nomenclature [24]. States including the H3K27ac mark, not covered in Roadmap, were designated using “active” (e.g., active enhancer, active transcription start site). For each cell type, chromatin states at the genomic position of measured CpGs were based on a majority call (same chromatin state in at least 3/5 donors); 5855 states were called in monocytes of the 5870 monocyte-to-macrophage differentiation DMCs, 5865 in macrophages. Enrichments for DMCs in chromatin states were calculated using Fisher’s exact test.

Promoter capture Hi-C interactions [16] were downloaded for monocytes and macrophages and enrichments were performed using Fisher’s exact test with *P*-values capped at 2.2 × 10^−16^, the lowest value that can be represented accurately [42].

Blueprint whole-genome bisulfite sequencing (WGBS) data were downloaded for monocytes (donors: C000S5, C0010K, C001UY and S007G7) and macrophages (donors: C005VG, S001S7, S0022I and S00390) [17] and methylation beta values were averaged for each cell type. DMRs were obtained using the following approach: the WGBS CpG overlapping with the DMC discovered in 450k data and each subsequent WGBS CpG both upstream or downstream was withing 1 Mb and had *a* ≥ 5% difference in methylation in the same direction.

Blueprint DNAseI hypersensitive sites sequencing peak files were downloaded and converted to a binary format (0/1) for monocytes (donors: C0010K, C0011I, C001UY, C00408, S00T4H, S00T5F, S00T6D, S00TA5, S00TT4, S00UKI, S00UME, S00YK2, S00YRP, S00YVH, S0100 M, S010B0, S010MF, S010P9, S010VY, S01238, S01246, S0130A, S01342, S0137X, S013CN and S013DL) and macrophages (donors: C005VG, C006UE, S001S7 and S0022I) [17]. For each cell type, a DNAseI hypersensitive site was called at a genomic position using majority call (≥ 13 in monocytes, ≥ 2 in macrophages). Enrichments were calculated using Fisher’s exact test with *P*-values capped at 2.2 × 10^−16^, the lowest value that can be represented accurately [42].

Motif analysis for transcription factor binding sites was performed using HOMER [25] with a 50 bp window around the DMCs. A random set of 50000 non-DMCs was used as a background. C/EBP and PU.1 ChIP-seq peak files [28] were downloaded for monocytes and macrophages, and enrichments were calculated using Fisher’s exact test for DMCs at motif locations.

## Additional files

**Additional file 1: Table S1** Antibodies used for flow cytometry analysis. **Figure S1**. Monocytes were successfully differentiated to macrophages. **Figure S2**. DNA methylation clusters on donor and monocyte versus macrophage. **Figure S3**. Distribution of beta values is generally uniform from ~0% to 100% methylation. **Figure S4**. There are 5 DMCs where the change in DNA methylation is contributed to more than one macrophage type. **Figure S5**. Differentially methylated CpGs were validated using public data. **Figure S6**. Transcription of genes was reduced near gain DMCs and increased near loss DMCs. **Figure S7**. Pathway analysis of LPS/IFNy macrophage-specific activation. **Figure S8**. Methylation differences for the differentially methylated CpGs were generally concordant with public WGBS data. **Figure S9**. Gain-DMC cg01059398, located in TNFSF10, is associated a DNAseI hypersensitive site and gain of PU.1 binding during monocyte-tomacrophage differentiation.

**Additional file 2: Table S2** Differentially methylated CpGs (DMCs). The table includes all 5870 DMCs with the characteristics for each position. Column 1: CpG identifier, Column 2-4: Effect size (mean squares), F-statistic and P-value of the overall effect, Column 5-10: Partial t-statistics for monocytes, macrophages and activated macrophages (LPS/IFNγ, IL-4, oxLDL and acLDL), Column 11: Cell type contributing most to the overall effect based on partial t-statistics, Column 12: Gain or loss of methylation during monocyte-to-macrophage differentiation or during subsequent macrophage activation, Column 13: Nearest gene, Column 14-15: Transcription levels of nearest gene in monocytes and macrophages (logTPM), Column 16-17: Chromatin states in monocytes and macrophages based on histone modifications, Column 18-20: Number, length (bp) and mean methylation (%) of differentially methylated CpGs in region (DMR), Column 21-22: Overlap with DNAseI hypersensitive sites in monocytes and macrophages.

**Additional file 3: Table S3** Chromatin states in monocytes and macrophages. Chromatin states in both monocytes and macrophages defined using a hidden Markov model based on H3K4me1, H3K4me3, H3K27ac and H3K27me3 histone marks available from public BLUEPRINT data.

## Data Availability

The DNA methylation dataset supporting the conclusions of this article is available in the EGA repository (EGAS00001003668). A workflow for the quality control and normalization pipeline is available at https://molepi.github.io/DNAmArray_workflow/index.html. Publicly available datasets were also used to support the conclusions of the article. Blueprint RNA-seq data [17] were downloaded for monocytes (donors: C000S5, C0010K, C0011I, C001UY and C004S) and macrophages (donors: C005VG, S001S7, S0022I and S00390) and transcription values (logTPM) were averaged for each cell type. Blueprint ChIP-seq peak files were downloaded for histon marks H3K4me1, H3K4me3, H3K27ac and H3K27me3 for 5 donors (C005VG, S001S7, S0022I, S00390 and S01F8K) with both monocyte and macrophage data [17]. Peak files were converted to a binary format (0 = no peak, 1 = peak). Blueprint whole-genome bisulfite sequencing (WGBS) data were downloaded for monocytes (donors: C000S5, C0010K, C001UY and S007G7) and macrophages (donors: C005VG, S001S7, S0022I and S00390) [17] and methylation beta values were averaged for each cell type. Blueprint DNAseI hypersensitive sites sequencing peak files were downloaded and converted to a binary format (0/1) for monocytes (donors: C0010K, C0011I, C001UY, C00408, S00T4H, S00T5F, S00T6D, S00TA5, S00TT4, S00UKI, S00UME, S00YK2, S00YRP, S00YVH, S0100 M, S010B0, S010MF, S010P9, S010VY, S01238, S01246, S0130A, S01342, S0137X, S013CN and S013DL) and macrophages (donors: C005VG, C006UE, S001S7 and S0022I) [17].
