# Supplementary material for: Human monocyte-to-macrophage differentiation involves highly localized gain and loss of DNA methylation at transcription factor binding sites
Source: Epigenetics Chromatin. 2019 Jun 6;12:34. doi: 10.1186/s13072-019-0279-4 (PMC6551876; doi:10.1186/s13072-019-0279-4)

Supplemental Tables

Supplemental Table S1: Antibodies used for flow cytometry analysis

Surface marker	Flurochrome	Dilution	Supplier
CD14	PE-Cy7	1/50	BD
CD16	APC-Cy7	1/50	BD
HLA-DR	PerCpCy5.5	1/50	BD
CCR5	FITC	1/10	BD
CD68	PE	1/50	BD
CCR7	PE	1/25	Biolegend
CD64	APC	1/50	Biolegend
CD200R	PE	1/25	Biolegend
CD206	FITC	1/25	Sony

Supplemental Figure Legends

Supplemental Figure S1: Monocytes were successfully differentiated to macrophages. (A) monocyte and macrophage purity based on CD14 and CD16 expression as assessed by flow cytometry. **(B)** Monocyte and macrophage purity (% CD14+, CD16+ cells) for the 4 donors and macrophage subsets. **(C)** surface expression of CD14, CD16, CD68, HLA-DR and CCR5 for monocytes and macrophage subsets. **(D)** Surface expression of the marker genes CCR7 and CD64 after stimulation with LPS (10ng/ml) + IFNy (50ng/ml) for all 4 donors. **(E)** Surface expression of the marker genes CD200R and CD206 after stimulation with IL-4 (50ng/ml) for all 4 donors. **(F)** Oil red O lipid staining for macrophages, oxLDL foam cells and acLDL foam cells.

Supplemental Figure S2: DNA methylation clusters on donor and monocyte versus macrophage. Principal components calculated on the methylation beta values for each sample. PC1 clusters on sex (37% variance explained), PC2-PC3 on donor (17% and 16% variance explained respectively) and PC4 clusters on monocyte versus macrophage (6% variance explained).

Supplemental Figure S3: Distribution of beta values is generally uniform from ~0% to 100% methylation. Difference in beta values for the 5780 monocyte-to-macrophage specific DMCs averaged for each donor for gain of methylation (red) and loss of methylation (blue).

Supplemental Figure S4: There are 5 DMCs where the change in DNA methylation is contributed to more than one macrophage type. Heatmap of partial t-statistics of DMCs for macrophages and activated macrophages reveals 5 DMCs where the change in DNA methylation is contributed to more than one macrophage type; cg04739200 (macrophage and M (IL−4)), cg27000690 (macrophage and M (IL−4)), cg06850284 (macrophage and M (acLDL)), cg26933866 (macrophage and M (LPS/IFNγ)) and cg23248885 (M (oxLDL)) and M (acLDL)).

Supplemental Figure S5: Differentially methylated CpGs were validated using public data. Cell type specific regression estimates obtained using a linear mixed model were compared for the 5870 differentially methylated CpGs with public data re-analyzed using the same method for monocytes, macrophages and LPS/IFNγ macrophages. Points are partly transparent to better capture differences in density.

Supplemental Figure S6: Transcription of genes was reduced near gain DMCs and increased near loss DMCs. Transcription of genes near DMCs for gain and loss of methylation for the pathways found in Figure 3A in monocytes and macrophages.

Supplemental Figure S7: Pathway analysis of LPS/IFNy macrophage-specific activation. Pathway analysis for GO-terms biological processes for differential DMCs mapped to their nearest gene for the 65 loss-DMCs during LPS/IFNγ macrophage-specific activation. Shown is the Top 10.

Supplemental Figure S8: Methylation differences for the differentially methylated CpGs were generally concordant with public WGBS data. Monocyte specific regression estimates obtained using a linear mixed model were compared for the 4648 differentially methylated CpGs with differences in monocyte and macrophage methylation in public WGBS data.

Supplemental Figure S9: Gain-DMC cg01059398, located in TNFSF10, is associated a DNAseI hypersensitive site and gain of PU.1 binding during monocyte-to-macrophage differentiation.
Visualization of chr3:172520017-172516017, 2000 bp up and downstream of cg01059398. Tracks from top to bottom: ENSEMBL genes, our 450k DNA methylation data, BLUEPRINT WGBS data (difference in macrophage and monocyte methylation), monocyte enhancers, macrophage enhancers, monocyte DNAseI hypersensitive sites, macrophage DNAseI hypersensitive sites, monocyte CEBP binding sites, macrophage CEBP binding sites, monocyte PU.1 binding sites, macrophage PU.1 binding sites.

Supplemental Figure S1

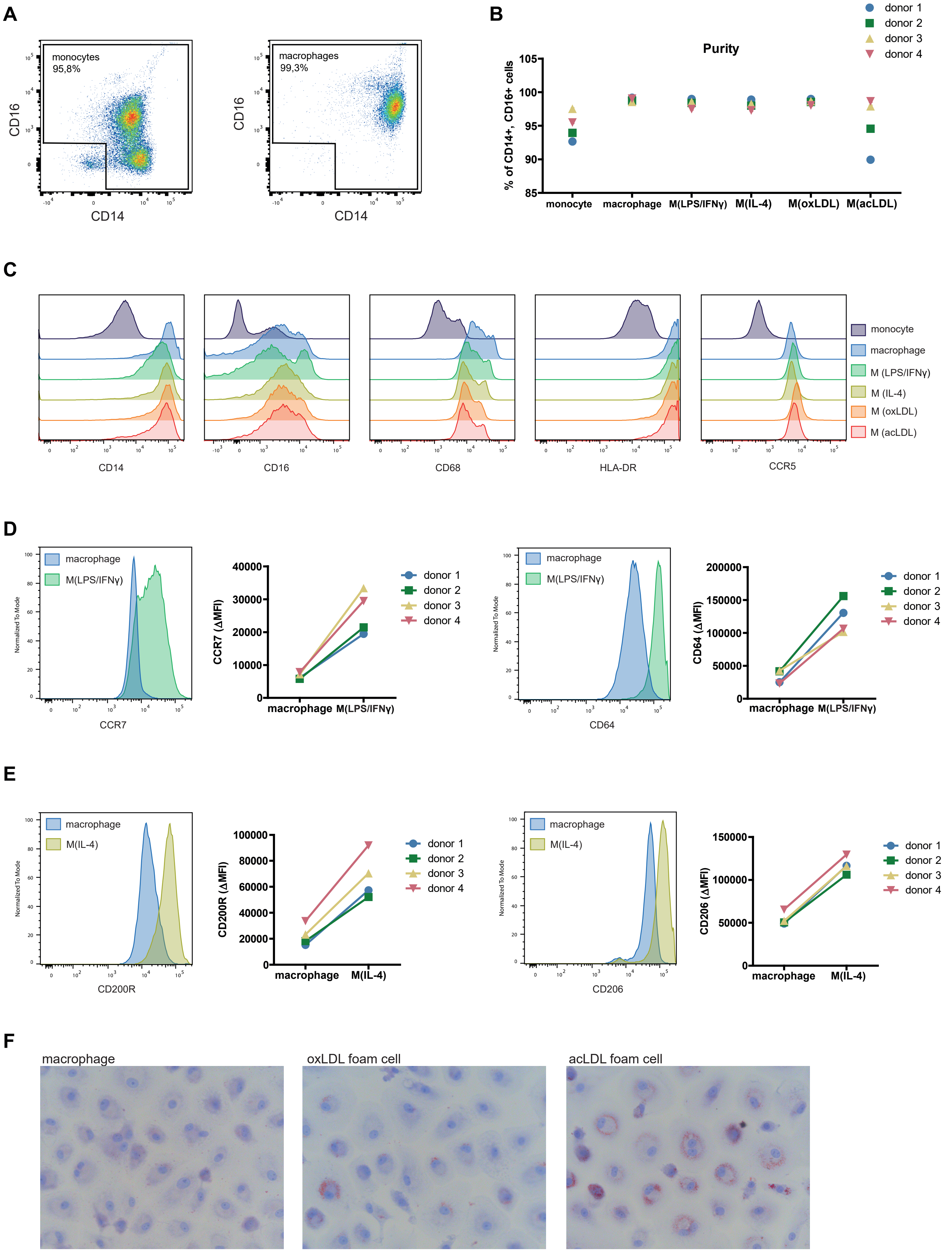

Supplemental Figure S2

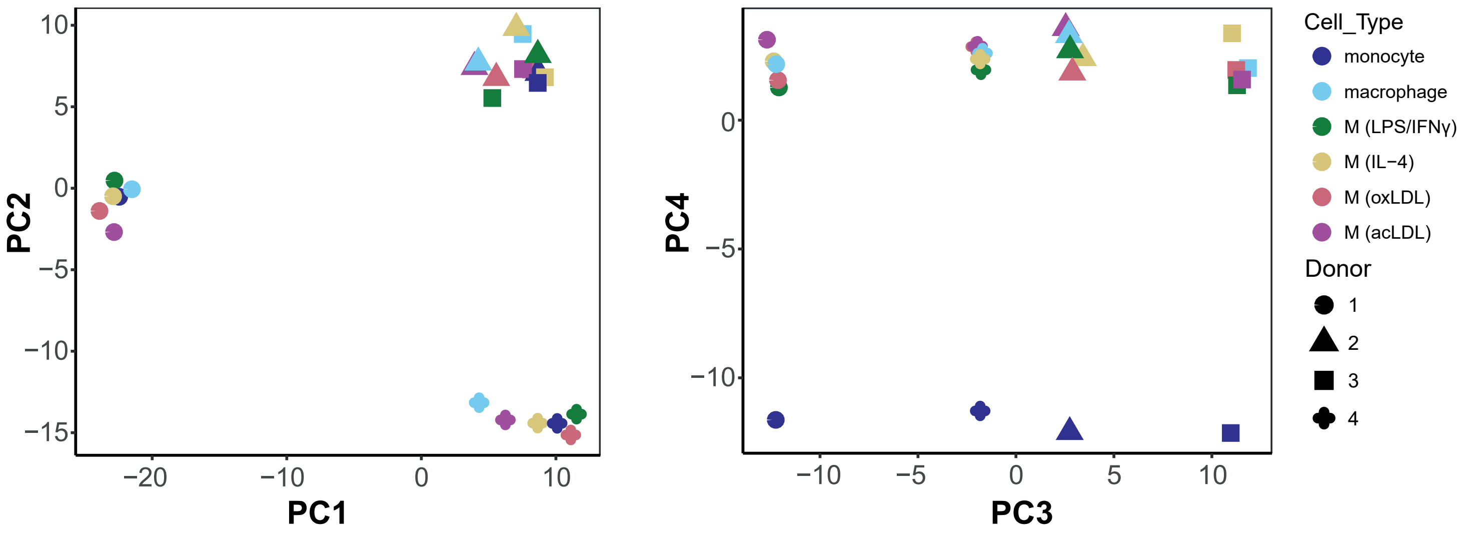

Supplemental Figure S3

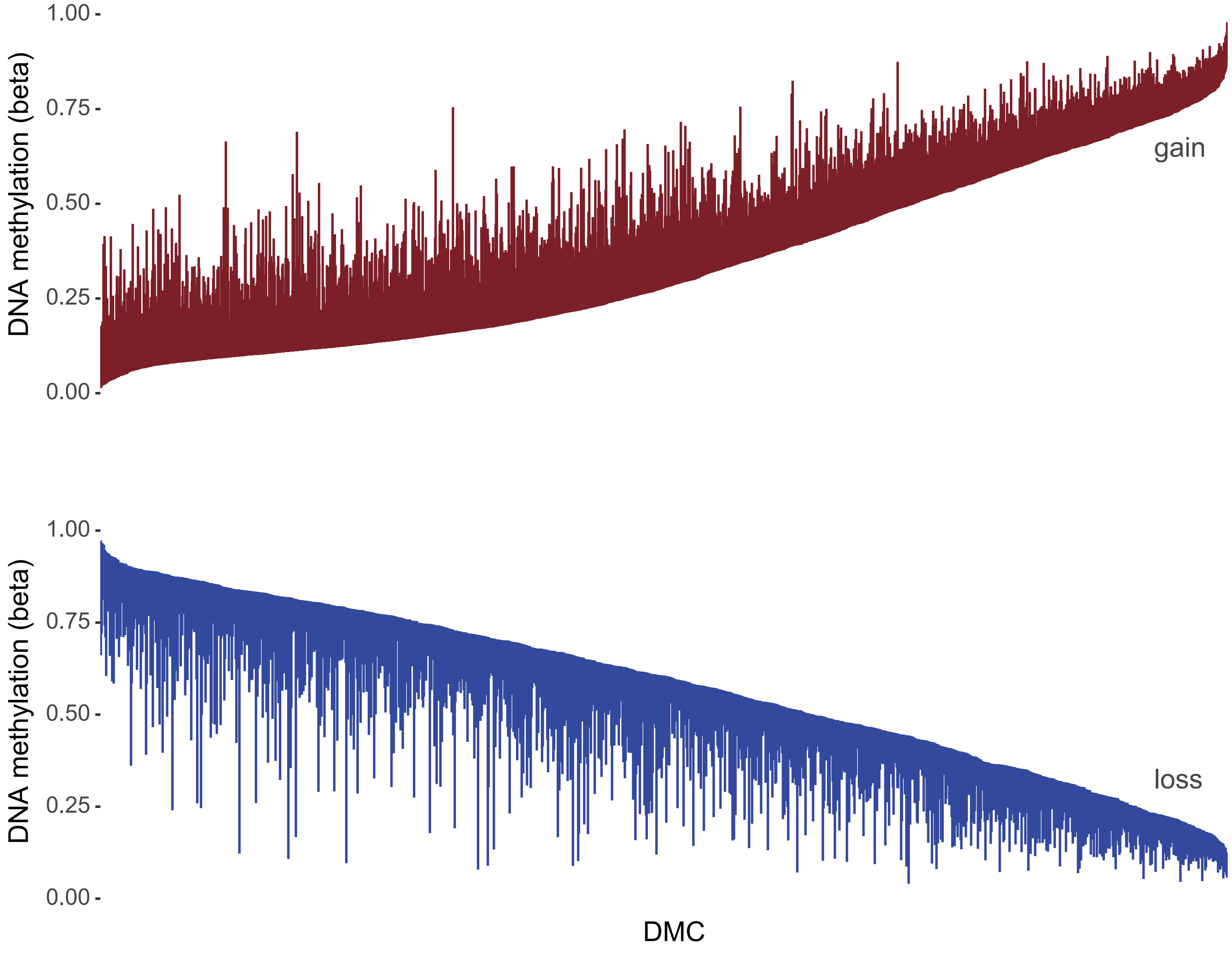

Supplemental Figure S4

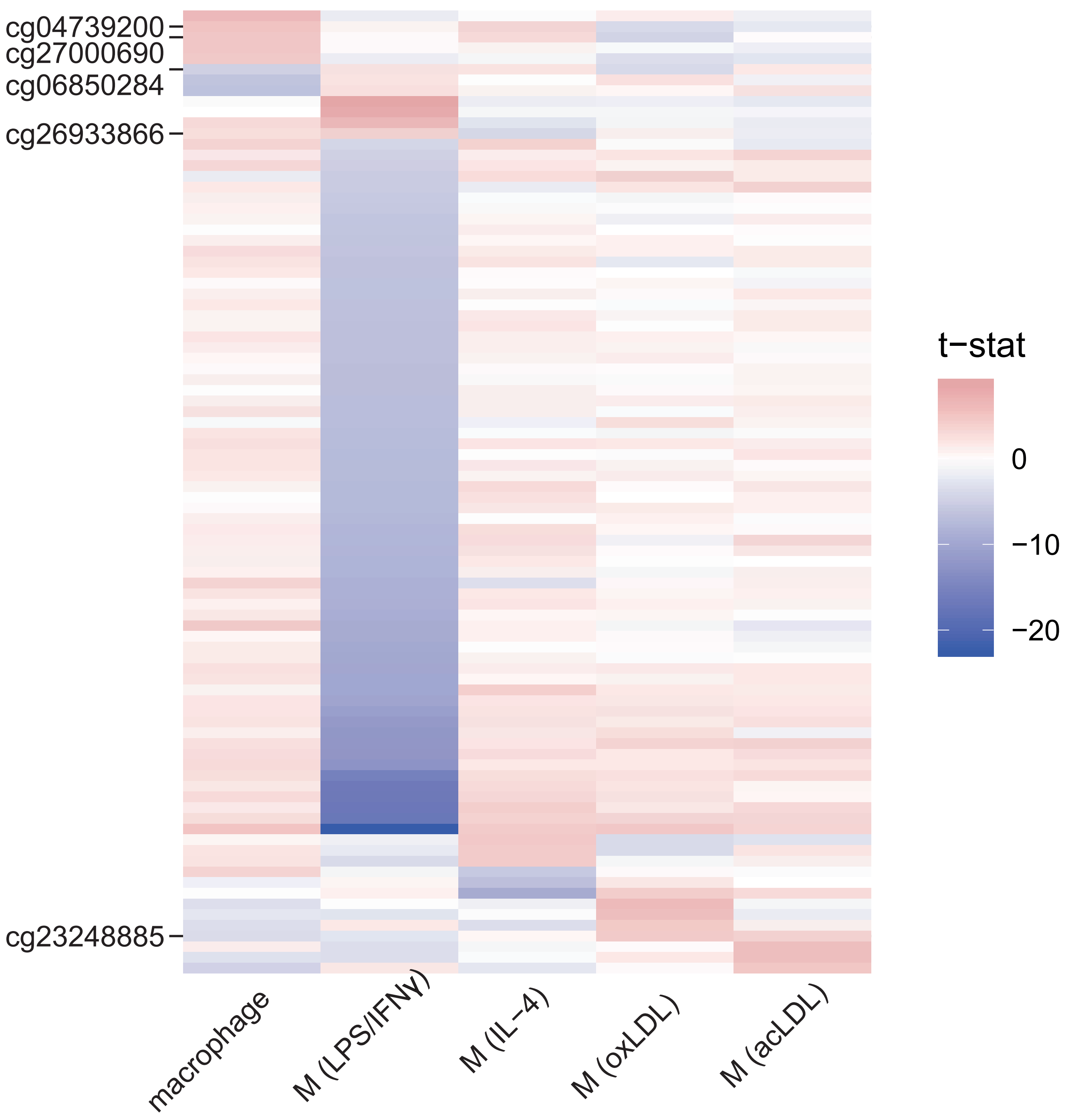

Supplemental Figure S5

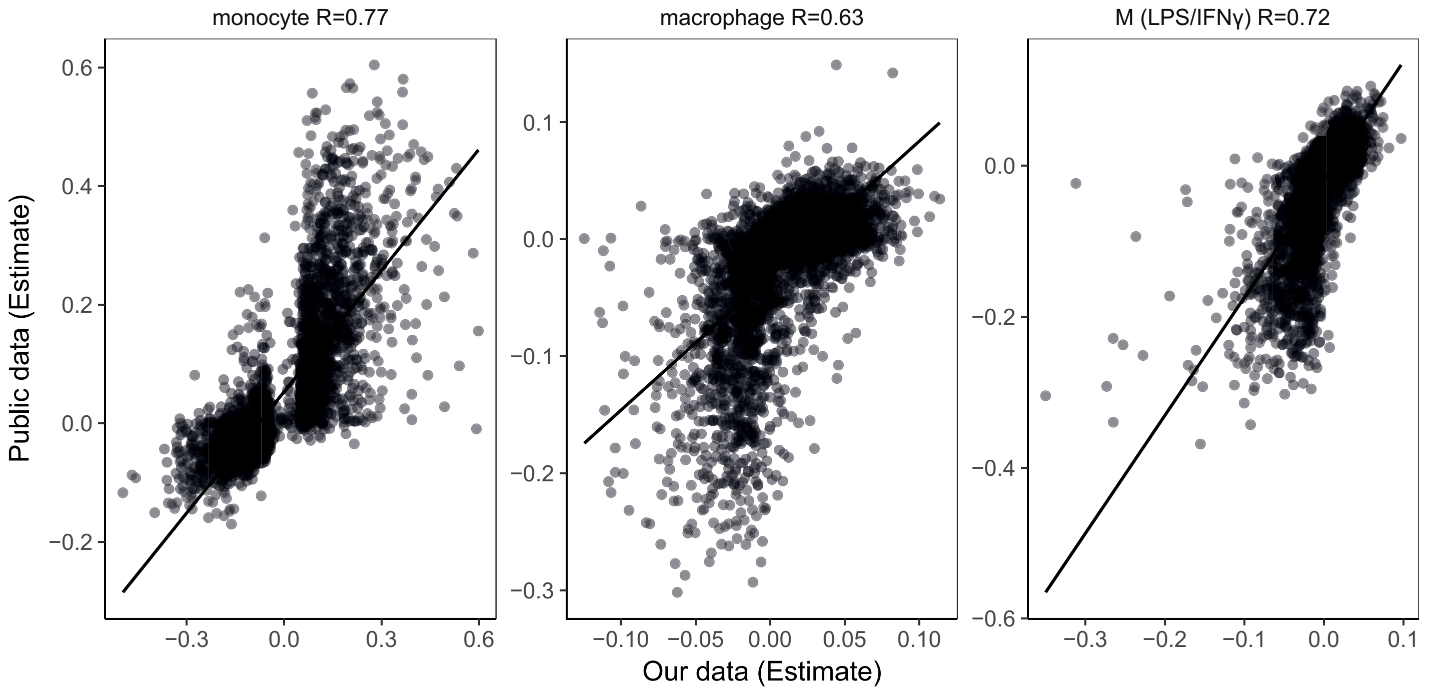

Supplemental Figure S6

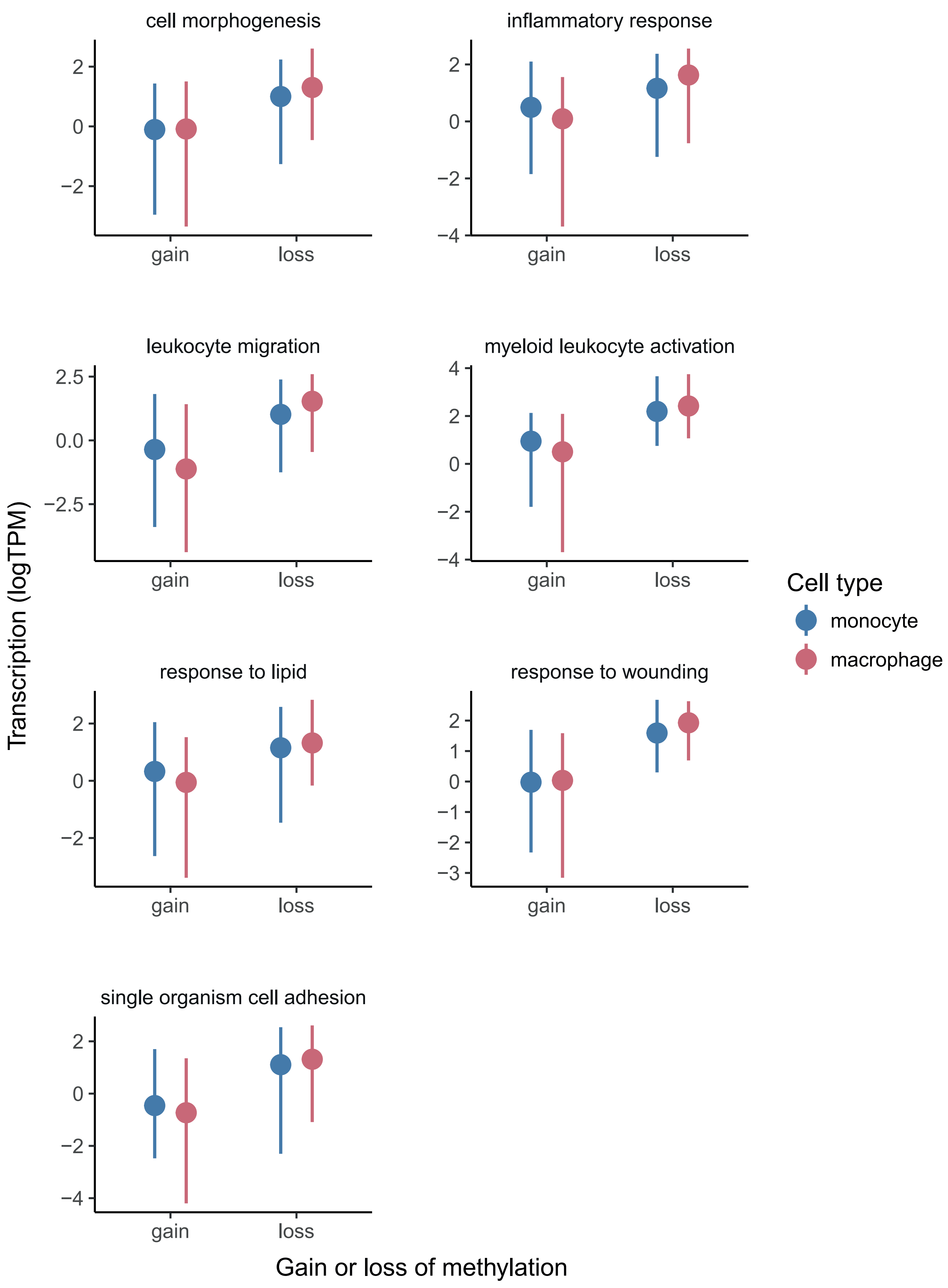

Supplemental Figure S7

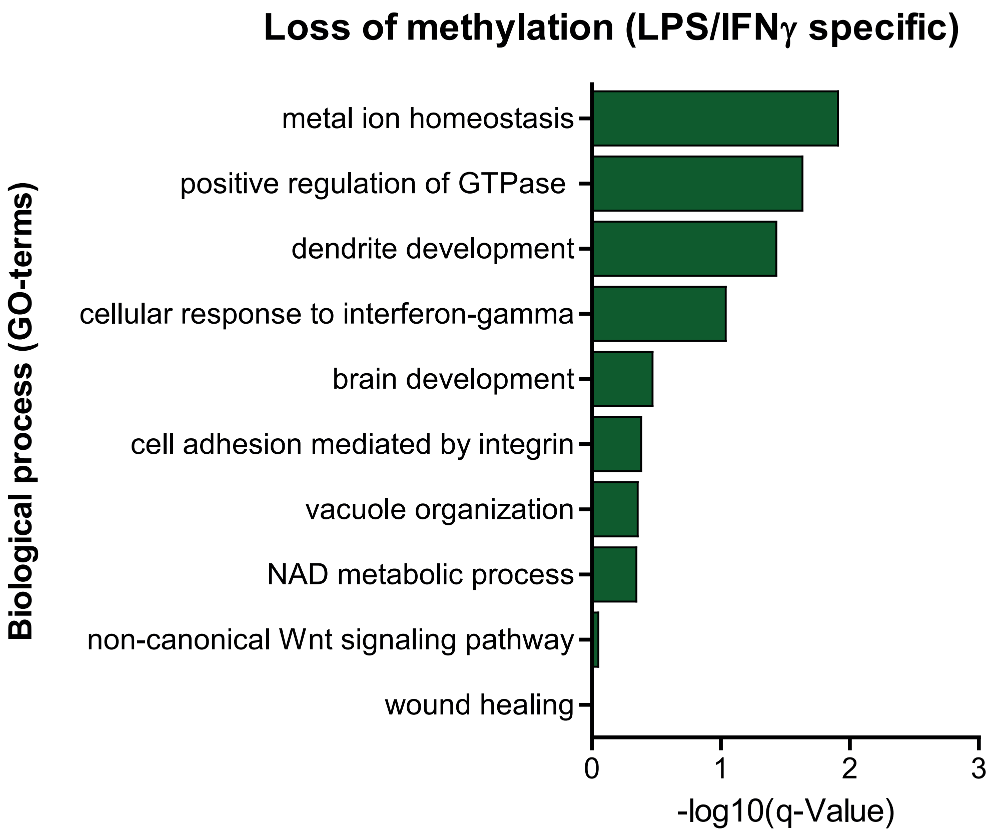

Supplemental Figure S8

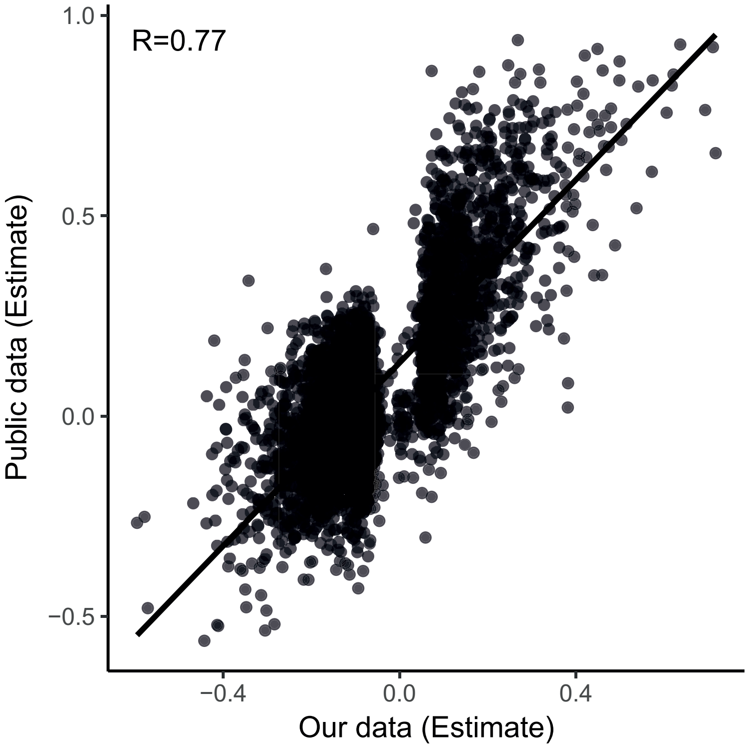

Supplemental Figure S9

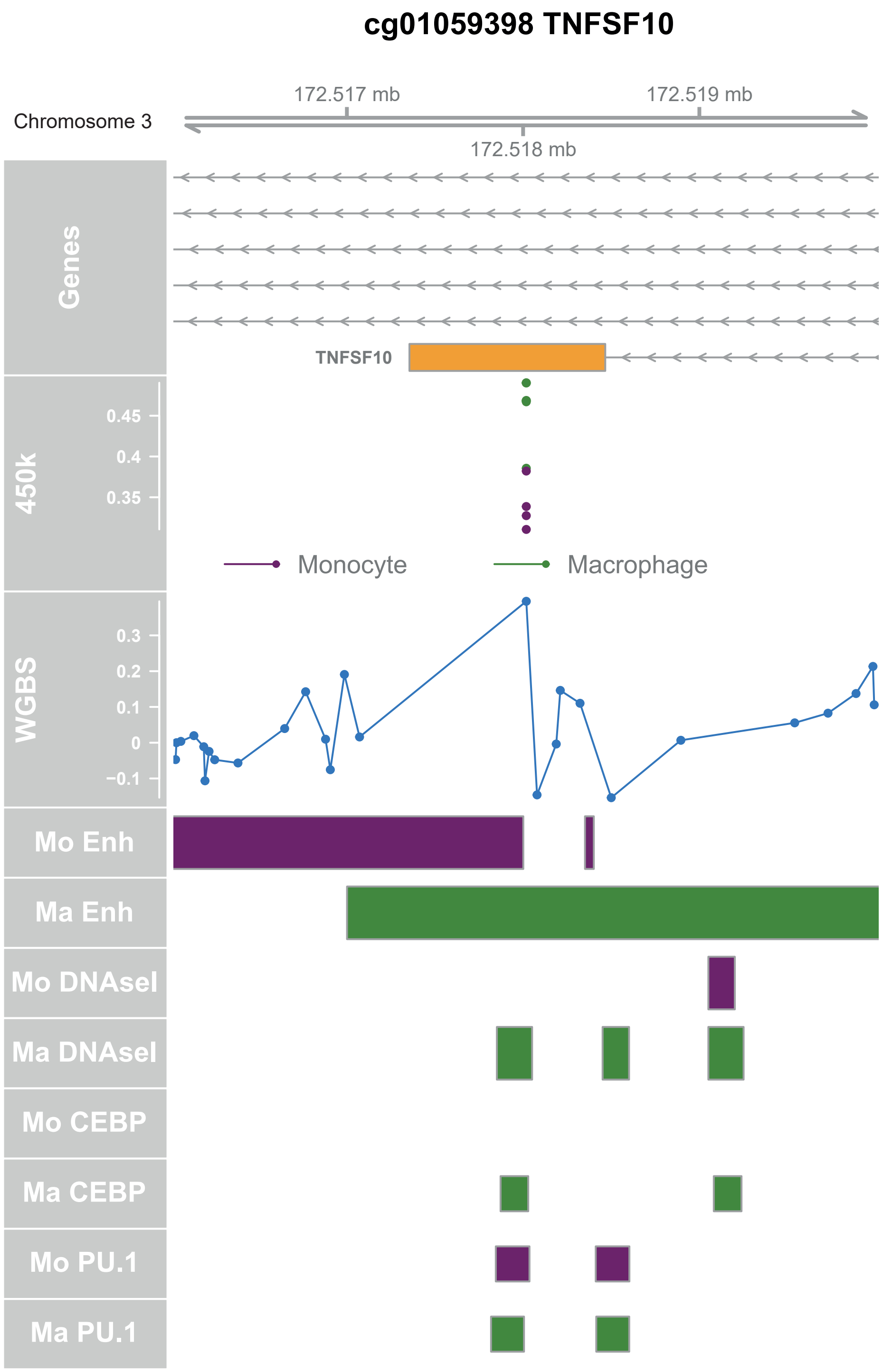

Supplement: Supplementary file 1 — Additional file 1: Table S1 Antibodies used for flow cytometry analysis. Figure S1. Monocytes were successfully differentiated to macrophages. Figure S2. DNA methylation clusters on donor and monocyte versus macrophage. Figure S3. Distribution of beta values is generally uniform from ~0% to 100% methylation. Figure S4. There are 5 DMCs where the change in DNA methylation is contributed to more than one macrophage type. Figure S5. Differentially methylated CpGs were validated using public data. Figure S6. Transcription of genes was reduced near gain DMCs and increased near loss DMCs. Figure S7. Pathway analysis of LPS/IFNy macrophage-specific activation. Figure S8. Methylation differences for the differentially methylated CpGs were generally concordant with public WGBS data. Figure S9. Gain-DMC cg01059398, located in TNFSF10, is associated a DNAseI hypersensitive site and gain of PU.1 binding during monocyte-tomacrophage differentiation. [file 13072_2019_279_MOESM1_ESM.pdf]
